# Clinical translation of human iPSC technologies: advances, safety concerns, and future directions

**DOI:** 10.3389/fcell.2025.1627149

**Published:** 2025-09-22

**Authors:** Sarah Dhaiban, Sanjana Chandran, Mohammed Noshi, Abdulrahim A. Sajini

**Affiliations:** ^1^ Department of Biological Sciences, Khalifa University of Science and Technology, Abu Dhabi, United Arab Emirates; ^2^ Department of Biomedical Engineering, Khalifa University of Science and Technology, Abu Dhabi, United Arab Emirates; ^3^ Sheikh Shakhbout Medical City, Abu Dhabi, United Arab Emirates; ^4^ Department of Biology, Chemistry, and Environmental Sciences, College of Arts and Sciences, American University of Sharjah, Sharjah, United Arab Emirates

**Keywords:** induced pluripotent stem cells (iPSCs), regenerative medicine, gene editing, clinical trials, HLA-matched iPSC banks, cell therapy, personalized medicine

## Abstract

Human induced pluripotent stem cells (hiPSCs) have opened new possibilities in regenerative medicine, providing a versatile platform for modeling human disorders, testing pharmacological agents, and developing personalized regenerative treatments. By reprogramming adult cells into a pluripotent state, scientists can generate patient-specific cells capable of differentiating into nearly any tissue type. Using the patient’s own cells allows for therapies that are both biologically matched and ethically acceptable, while also reducing the likelihood that the immune system will reject transplanted cells. Despite this promise, translating hiPSCs into routine clinical use has proven challenging, with several practical and biological barriers yet to be overcome. Key concerns include variability in differentiation outcomes, immune responses to allogeneic cells, genetic and epigenetic abnormalities, and the risk of tumor formation. Reliable scale-up under GMP conditions remains a major technical hurdle, and critical questions around long-term engraftment, tissue integration, and immune tolerance are still unresolved. Recent advances, including CRISPR/Cas9 gene editing and AI-guided differentiation, are enhancing iPSC quality and enabling treatments to be tailored to individual patients. Clinical trials are ongoing in areas such as retinal disorders, neurodegenerative diseases, cardiac conditions, and cancer immunotherapy, with early findings suggesting these therapies may be both feasible and safe. However, widespread adoption will require rigorous, long-term evaluation. This review examines the latest progress in hiPSC technology and evaluates its movement toward clinical translation. We highlight the major challenges that continue to limit broader application, particularly those related to safety, large-scale manufacturing, and regulatory oversight, and discuss emerging advances that may help bring iPSC-based therapies closer to routine clinical practice.

## Graphical Abstract

**Figure:**
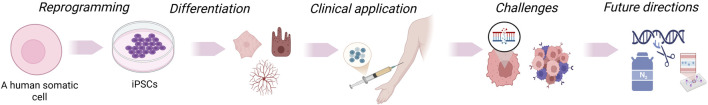
Somatic cells are reprogrammed into induced pluripotent stem cells (iPSCs), which can be differentiated into various cell types. These cells may be genetically modified, expanded, and utilized in disease modeling, drug testing, and transplantation. iPSC-based approaches enable regenerative therapies, immune engineering, and personalized medicine, though challenges remain before clinical translation.

## 1 Introduction

The discovery of induced pluripotent stem cells (iPSCs) by Takahashi and Yamanaka (2006) marked a transformative milestone in regenerative medicine, demonstrating that adult somatic cells could be reprogrammed into pluripotent stem cells using four transcription factors (Takahashi and Yamanaka, 2006). This achievement was extended to human cells later, generating patient-specific iPSCs from adult fibroblasts (Takahashi et al., 2007). Crucially, this method circumvents ethical controversies linked to embryonic stem cells (Denker, 2006).

iPSCs have enabled the creation of disease-specific cellular models for conditions such as Parkinson’s disease, Alzheimer’s disease, Duchenne muscular dystrophy, and type I diabetes, facilitating patient-specific mechanistic studies and therapeutic screening (Park et al., 2008a; Soldner et al., 2009; Jang et al., 2012). More recently, Tanaka et al. (2015) used iPSC-derived cardiomyocytes to replicate inherited arrhythmias, confirming their utility in functional drug testing (Tanaka et al., 2015).

Over the past year, the field has seen unprecedented clinical advances. A Phase I/II trial published in April 2025 reported that allogeneic iPSC-derived dopaminergic progenitors survived transplantation, produced dopamine, and did not form tumors in Parkinson’s patients (jRCT2090220384) (Sawamoto et al., 2025).

Concurrently, an ongoing autologous iPSC-derived dopamine neuron trial at Mass General Brigham is pioneering the use of a patient’s own blood-derived iPSCs in Parkinson’s disease, eliminating the need for immune suppression (HPSC, 2024).

In the retinal field, Eyecyte-RPE, an iPSC-derived RPE product, received IND approval in India in 2024 for geographic atrophy associated with AMD, an important step toward scalable and cost-effective cell therapy approaches (Soundararajan et al., 2025).

Yet significant challenges remain. Recent preclinical development of clinical-grade iPSC lines from Parkinson’s patients revealed ongoing concerns related to genomic stability and cell line quality control (Jeon et al., 2025). In non-human primates, iPSC-derived cardiomyocyte patches improved cardiac performance but induced transient arrhythmias, which indicates the safety and scalability challenges in cardiac applications (Shiba et al., 2016).

CRISPR-Cas9 genome editing has become an essential tool in iPSC-based disease modeling and therapeutic development. In Parkinson’s disease, for example, Soldner et al. (2011) used CRISPR to correct the A53T *SNCA* mutation in patient-derived iPSCs, creating isogenic lines for mechanistic studies (Soldner et al., 2011). In a more recent study, Chang et al. (2021) used CRISPR to edit iPSCs from Parkinson’s patients carrying *LRRK2* and *PARK2* mutations. After correction, the neurons exhibited improved mitochondrial activity and more intact nuclear envelopes, underscoring how gene editing can sharpen the accuracy and usefulness of iPSC models in studying disease and exploring treatment strategies (Chang et al., 2021). Meanwhile, new AI and machine learning methodologies, such as automated colony morphology classification and differentiation outcome prediction, are being applied to enhance standardization, quality control, and reproducibility in iPSC manufacturing (Vedeneeva et al., 2023).

While many previous reviews have focused on specific technical aspects, such as reprogramming strategies, disease modeling, or immune modulation, few have brought together the latest clinical trials, manufacturing practices, safety data, and enabling technologies into a single, integrated analysis. In this review, we bring together the latest progress in iPSC-based therapies, with a focus on clinical applications, regulatory developments, and new enabling technologies. Our aim is to offer a useful and forward-thinking resource for researchers, clinicians, and policymakers working to advance the safe translation of iPSC innovations into medical practice.

## 2 Historical perspective and mechanistic foundations of iPSC technology

The ability to reprogram adult somatic cells into a pluripotent state build on decades of foundational work in developmental biology. In 1952, Briggs and King demonstrated that embryonic nuclei could support development when transferred into enucleated amphibian eggs, laying the groundwork for somatic cell nuclear transfer (SCNT) (Briggs and King, 1952). A decade later, Gurdon provided direct evidence of cellular plasticity by reprogramming differentiated intestinal epithelial cells to an embryonic state using SCNT (Gurdon, 1962).

These early discoveries paved the way for the derivation of embryonic stem cells (ESCs) from mouse blastocysts in 1981 (Evans and Kaufman, 1981; Martin, 1981). And eventually led to the birth of Dolly the sheep in 1996, the first animal cloned from an adult somatic cell (Campbell et al., 1996; Wilmut et al., 1997). In 1998, human ESCs were derived from blastocyst-stage embryos (Thomson et al., 1998), but their use raised ethical and immunological concerns that prompted the search for alternative pluripotent cell sources.

**FIGURE 1:**
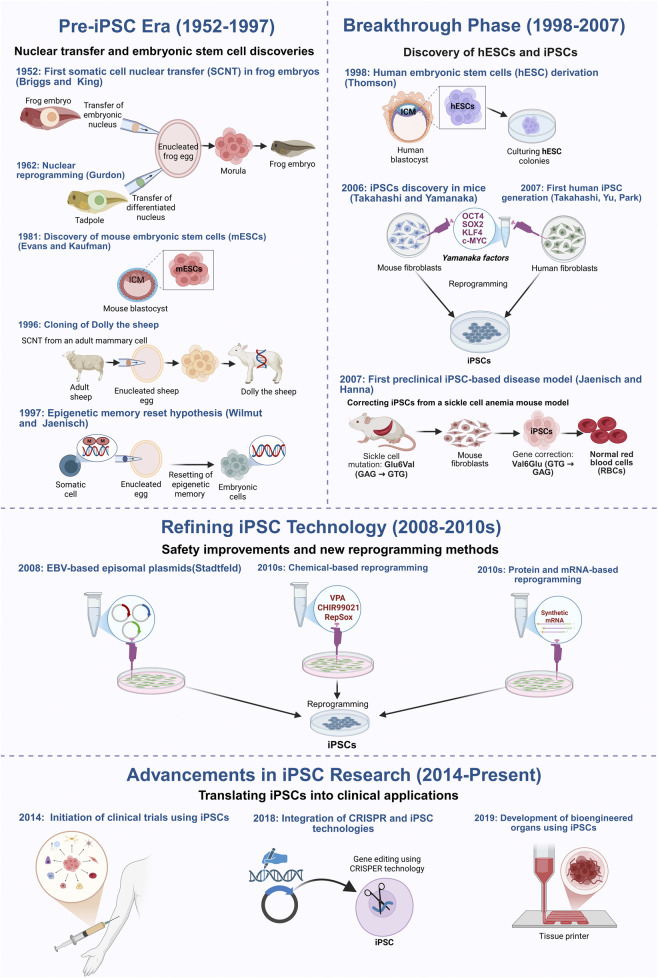
Key milestones in the development of induced pluripotent stem cells (iPSCs) technology and the different reprogramming methods. This figure outlines the key milestones in the development of induced pluripotent stem cell (iPSC) technology, starting with the early foundational nuclear reprogramming experiments, the groundbreaking discovery of reprogramming factors, and the current ongoing research that aims at improving the safety and clinical application of iPSCs. The timeline begins in 1952, when Briggs and King first demonstrated somatic cell nuclear transfer (SCNT) in amphibians, proving that embryonic nuclei retain the ability to direct development. This was further validated by John Gurdon, 1962, who successfully reprogrammed differentiated cells into an embryonic state, establishing the reversibility of cellular identity. The isolation of mouse embryonic stem cells (mESCs) in 1981 confirmed the existence of *in vitro* pluripotent cells, while the cloning of Dolly the sheep in 1996 demonstrated that somatic cells could be reprogrammed despite epigenetic modifications. The discovery of human embryonic stem cells (hESCs) in 1998 paved the way for regenerative medicine but raised ethical concerns, driving efforts to develop alternative sources of patient-specific pluripotent cells. In 2006, Takahashi and Yamanaka identified four transcription factors (OSKM) capable of reprogramming mouse fibroblasts into iPSCs, a breakthrough extended to human cells in 2007. That same year, Jaenisch and Hanna developed the first iPSC-based preclinical disease model, demonstrating gene correction in sickle cell anemia. Subsequent research focused on improving iPSC safety and efficiency by replacing viral vectors with non-integrating methods, including adenoviral vectors (2008), plasmid-based transfection, direct protein/mRNA delivery, and chemical reprogramming. More recently, CRISPR-Cas9 technology has further enhanced precise genetic correction, while advances in bioengineered tissues and organoids have demonstrated the potential of iPSCs for generating functional tissues for transplantation. Figure generated using BioRender.com.

A major breakthrough came in 2006 when Takahashi and Yamanaka identified four transcription factors, *OCT4*, *SOX2*, *KLF4*, and *c-MYC* (*OSKM*), capable of reprogramming mouse fibroblasts into pluripotent cells (Takahashi and Yamanaka, 2006). This method was quickly adapted for human cells using retroviral and lentiviral systems, giving rise to induced pluripotent stem cells (iPSCs) that closely resemble ESCs in gene expression and differentiation potential, without the ethical limitations of embryo-derived cells (Takahashi et al., 2007; Park et al., 2008b; Scesa et al., 2021). Figure 1 summarizes these pivotal milestones, tracing the evolution of reprogramming from early nuclear transfer experiments to the emergence of iPSC-based therapies now entering clinical trials.

**FIGURE 2:**
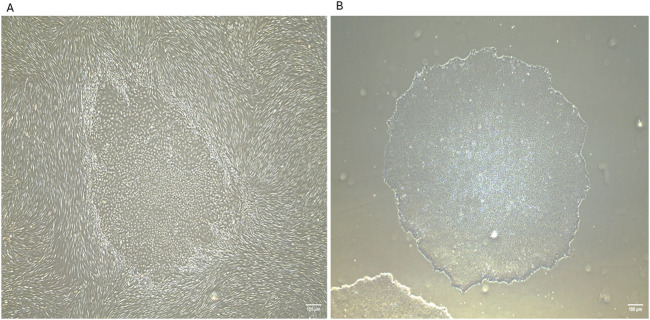
Generation of human induced pluripotent stem cells (iPSCs) from neonatal human dermal fibroblasts using Sendai reprogramming vectors. **(A)** iPSC colonies emerging within the fibroblast culture at day 16 post-reprogramming from human neonatal dermal fibroblasts (HDFn), displaying distinct morphology with tightly packed cells and defined borders. **(B)** Isolated iPSC colony at passage 2, exhibiting characteristic pluripotent stem cell morphology and colony structure. IPSCs were generated in the laboratory of Prof. Abdulrahim Sajini at Khalifa University of Science and Technology. These images were generated in-house by the authors and have not been previously published. Images were captured using an EVOS microscope with a ×4 objective lens. Scale bar = 100 µm.

Interestingly, early iPSC reprogramming strategies raised safety concerns due to the use of integrating viral vectors, which could disrupt host genomes and increase tumorigenic risk. This prompted the development of safer, non-integrating methods, including adenoviral vectors (Stadtfeld et al., 2008), episomal plasmids (Yu et al., 2009), synthetic mRNAs (Warren et al., 2010), and Sendai virus vectors (Fusaki et al., 2009) (Figure 2). The therapeutic potential of iPSCs was first demonstrated in a 2007 study that corrected a sickle cell mutation in a mouse model (Hanna et al., 2007), establishing proof-of-concept for genetic repair using reprogrammed cells.

Mechanistically, reprogramming involves extensive transcriptional and epigenetic remodeling. It generally occurs in two phases: an early phase in which somatic identity is suppressed, and a late phase characterized by the stabilization of the pluripotency network (Buganim et al., 2013; Apostolou and Hochedlinger, 2013). Initially, chromatin is largely inaccessible to OSKM factors but gradually becomes more permissive as pluripotency genes are activated (Li et al., 2010; Soufi et al., 2012).

Epigenetic resetting is central to this process. Activating histone marks like H3K4me3 are enriched at pluripotency loci, while repressive marks such as H3K27me3 are reduced (Soufi et al., 2012). *SOX2* facilitates chromatin opening and demethylation (Zaret and Carroll, 2011), while *TET* enzymes, enhanced by vitamin C, promote DNA demethylation at key regulatory genes like *OCT4* (Blaschke et al., 2013; Habibi et al., 2013). Chromatin remodelers, including the SWI/SNF complex, reposition nucleosomes to enable transcription factor binding (Zaret and Carroll, 2011; Ho et al., 2009). Noncoding RNAs also contribute: long noncoding RNAs recruit chromatin modifiers (Loewer et al., 2010), and microRNAs like *miR-302* and *miR-145* regulate gene networks that govern pluripotency and *differentiation* (Kuppusamy et al., 2015). In parallel, signaling pathways such as BMP, *Wnt*, and *TGF-β* modulate transitions like the mesenchymal-to-epithelial transition (MET), which is essential for reprogramming success (Pasque et al., 2014).

Supplementary Table S1 provides a comparative summary of the major reprogramming approaches used to generate iPSCs. It outlines their integration profiles, efficiencies, timelines for colony emergence, key advantages, and limitations, helping contextualize each method in terms of safety and translational potential.

To address clinical safety concerns, non-integrating and chemically defined reprogramming systems have gained traction. Small molecules such as CHIR99021 (a GSK3β inhibitor) and valproic acid (a histone deacetylase inhibitor) have been shown to improve reprogramming efficiency by influencing metabolic activity and chromatin structure (Huangfu et al., 2008; Li et al., 2009). Researchers have used high-throughput screening and single-cell RNA sequencing to identify blocks in reprogramming and adjust experimental conditions CRISPR/Cas9 has been used to modify epigenetic regulators and increase consistency in reprogrammed cell populations (Kearns et al., 2015; Liu et al., 2016; Meng et al., 2020).

In parallel, bioengineering advances, including 3D organoids and biomimetic scaffolds, are creating more physiologically relevant environments for reprogramming and differentiation (Han et al., 2013; Caiazzo et al., 2016). Automation and robotics are also improving scalability and reproducibility in iPSC workflows (Paull et al., 2015; Tristan et al., 2020). Induced multipotent stem cells (iMSCs) have recently been developed as an alternative to traditional MSCs. They show broader differentiation capacity and a lower risk of tumor formation (Buitrago et al., 2024; Wu Z. et al., 2024).

As a result, iPSC-based strategies are now entering early clinical applications in several fields. Refinements in protocols and clearer regulatory guidance are making both autologous and allogeneic iPSC therapies more practical to deliver in clinical settings.

## 3 Current iPSC-based therapies

With the continuous progress in clinical translation, iPSC-based therapies are now being actively explored across a range of diseases. The following sections highlight key therapeutic areas where iPSCs have shown the most clinically promising studies to date.

### 3.1 Clinical applications of iPSC in AMD and retinal therapies

Ophthalmic applications of iPSCs have progressed significantly, with particular emphasis on retinal disorders such as age-related macular degeneration (AMD) (Tsai et al., 2015; Fields et al., 2016). iPSCs can be differentiated into retinal pigment epithelium (RPE) cells and photoreceptors, 2 cell types that are critical for normal visual function (Garcia et al., 2015; Hazim et al., 2017; Dehghan et al., 2022). In AMD, loss of RPE cells disrupts photoreceptor function and leads to vision loss (De et al., 2007; Zhang et al., 2021). Preclinical studies indicate that subretinal delivery of iPSC-derived RPE cells can protect or restore retinal function (Tokuyama et al., 2021).

Clinical translation has already begun. In Japan, autologous iPSC-derived RPE cells were transplanted into a patient with exudative AMD, and the graft remained stable without major complications (Mandai et al., 2017). More recently, Soma et al. (2024) showed that iPSC-derived corneal epithelium could also be engrafted safely in humans (Soma et al., 2024).

Donor-derived iPSCs are being investigated as an allogeneic, “off-the-shelf” source of RPE cells, while photoreceptor replacement is under investigation for advanced retinal disease (Maeda and Takahashi, 2023).

For instance, transplanted human iPSC-derived photoreceptors, when placed into cone-dominant ground squirrels, survived for 4 months but exhibited poor integration and no functional recovery (Yu et al., 2024). In Pde6b knockout rats, grafts survived longer, maintained visual responses, and showed no abnormal growth (Yang et al., 2021). Zhao et al. (2024) used chemically induced pluripotent stem cells (CiPSCs) in mice and achieved retinal integration and some functional rescue (Zhao et al., 2024).

Ensuring long-term safety is still a central challenge. GMP-grade iPSC-derived RPE cells did not form tumors in immunodeficient rodents, but abnormal proliferation is still possible (Zhang et al., 2021). CRISPR-modified MHC-II-deficient RPE cells survived in non-human primates without signs of inflammation (Ishida et al., 2024). Other editing strategies are being developed to remove oncogenic sequences and improve safety (Martin et al., 2020).

Clinical trials are now testing whether iPSC-derived RPE and photoreceptor grafts can provide lasting functional recovery in AMD and other retinal diseases (Liu et al., 2024).

### 3.2 Clinical applications of iPSC in neurodegenerative diseases treatment

Beyond ophthalmology, patient-derived iPSCs are increasingly applied to model neurodegenerative diseases such as Parkinson’s disease and ALS (Soldner et al., 2009; Fujimori et al., 2018). These models enable the study of disease mechanisms in a patient-specific setting and provide platforms for testing therapeutic strategies. Neurons generated from patient iPSCs have been used to reproduce disease phenotypes *in vitro* and to evaluate candidate interventions (Antoniou et al., 2022).

Parkinson’s disease has received particular attention. This disorder is defined by the progressive loss of dopaminergic neurons in the substantia nigra, which results in motor decline. Patient-derived iPSCs can be differentiated into dopaminergic neurons. These cells allow researchers to investigate disease mechanisms and explore cell replacement therapies (Doi et al., 2020). In preclinical models, transplantation of these neurons has restored dopamine levels and improved motor symptoms (Song et al., 2020; Morizane, 2023). Building on these findings, clinical studies are ongoing to examine the safety and potential efficacy of iPSC-based therapies in patients (Sugai et al., 2021). One notable case report described clinical improvement within 18–24 months following autologous transplantation of iPSC-derived dopaminergic progenitors (Schweitzer et al., 2020).

### 3.3 iPSC-derived immune cells for cancer therapy

iPSC technology has enabled large-scale production of immune cells, opening new avenues for cancer immunotherapy (Zhou et al., 2022). NK cells derived from iPSCs are particularly valuable because they can eliminate malignant cells without prior sensitization. When engineered with chimeric antigen receptors (CARs), these NK cells acquire enhanced specificity and cytotoxicity toward tumor cells (Li et al., 2018). A prominent example is FT596, an allogeneic CAR-NK product generated from iPSCs, which has advanced into clinical trials in the United States. This therapy incorporates an anti-CD19 CAR to improve the persistence and activity of NK cells *in vivo* (Ghobadi et al., 2025).

iPSC-derived T cells are also under investigation for adoptive immunotherapy. These cells can be engineered to carry tumor-specific receptors, enabling selective targeting of cancer cells (Themeli et al., 2013). Studies have demonstrated that iPSC-derived T cells display strong anti-tumor activity and sustained survival *in vivo* (Kawamoto et al., 2021; Cichocki et al., 2023). More recently, a feeder-free approach that inhibits G9a/GLP histone methyltransferases has been developed, yielding populations that closely resemble mature αβ T cells (Jing et al., 2024).

In addition, dendritic cells (DCs) generated from iPSCs are being explored for cancer vaccination. By loading these DCs with tumor antigens, they can be used to prime the immune system against specific malignancies (Mellman and Steinman, 2001; Calmeiro et al., 2020; Oba et al., 2021). Preclinical studies have shown that iPSC-derived DCs can trigger robust tumor-specific immune responses, demonstrating their potential as personalized immunotherapy strategy (Ackermann et al., 2020; Calmeiro et al., 2020; Oba et al., 2021).

Despite these advances, translation of iPSC-derived immune cells into the clinic continues to face challenges, including genomic instability, the potential for tumor formation, and variability in differentiation outcomes (Madrid et al., 2024). Addressing these concerns requires refinement of reprogramming and differentiation methods alongside the introduction of strict quality control standards to ensure clinical safety (Utikal et al., 2009; He et al., 2023). Ongoing efforts aim to resolve these limitations and advance their clinical use (Fang et al., 2025).

### 3.4 IPSC-derived cardiomyocyte sheets for treatment of heart failure

iPSC-derived cardiomyocytes are being investigated for cardiac repair (Kawamura et al., 2023). Shiba et al. (2016) were among the first to show that transplanting iPSC-derived cardiomyocyte patches into primates could support myocardial regeneration, although transient arrhythmias were noted in some animals (Shiba et al., 2016).

Miyagawa et al. (2022) later used clinical-grade, HLA-homozygous hiPSC-derived cardiomyocytes in a porcine model and observed improved cardiac function and angiogenesis, with no evidence of tumors, genetic abnormalities, or arrhythmias. A study by Jebran et al. (2025) involved transplanting engineered heart muscle composed of iPSC-derived cardiomyocytes and stromal cells into rhesus macaques with chronic heart failure. The grafts improved contractility and remained stable for several months, without evidence of arrhythmias or tumor formation. These findings supported a first-in-human implantation, which demonstrated graft survival and structural remuscularization in a patient with advanced heart failure (Jebran et al., 2025).

Early clinical trials have now begun evaluating cardiomyocyte sheets in patients (Kawamura et al., 2023). In one case, a patient with ischemic cardiomyopathy received an iPSC-derived cardiomyocyte patch and showed improved cardiac function 6 months after surgery, with no major complications (Miyagawa et al., 2022). Still, several challenges remain, particularly achieving long-term cell survival, stable electrical integration, and scalable, consistent production. Current efforts aim to improve cell maturation, reduce arrhythmogenic risk, and refine GMP-compliant manufacturing protocols (Silver et al., 2021; Jiang et al., 2024; Raniga et al., 2024).

An overview of clinical trials involving iPSC-based therapies across multiple indications is provided in Table 1.

**TABLE 1:** Summary of clinical trials involving induced pluripotent stem cell (iPSC)-based therapies. The table outlines trial name and institution, iPSC-derived cell source, target indication, clinical trial phase, outcome summary, current status, year of initiation and/or completion, and reference. Trials span cardiology, endocrinology, hematology, neurology, ophthalmology, oncology, and orthopedics. All identifiers (e.g., NCT, jRCT, ChiCTR, UMIN) are sourced from official registries. Interventional studies are listed unless otherwise noted (e.g., “Observational”).

System/Indication area	Trial name/Institution	Cell source	Target indication	Phase	Outcome summary	Current status	Year started/Completion	References
Cardiology	BioVAT-HF - University Medical Center Göttingen (NCT04396899)	iPSC-derived cardiomyocytes + stromal cells (EHM)	Advanced heart failure (EF ≤ 35%)	Phase I/II	Biological ventricular assist tissue (BioVAT) implanted to promote myocardial remuscularization	Ongoing	2020/2027 (est.) (Germany)	https://clinicaltrials.gov/study/NCT04396899
	iPSC-CM Patch - Kyoto/Osaka Univ. (jRCT2053190081)	Allogeneic iPSC-derived cardiomyocyte sheets	Ischemic cardiomyopathy	Phase I	First patient showed improved wall motion, no major AEs at 6 months 10 patients planned	Not recruiting; follow-up ongoing	2020/- (Japan)	Miyagawa et al. (2022) https://trialsearch.who.int/Trial2.aspx?TrialID=JPRN-jRCT2053190081 (https://jrct.niph.go.jp/latest-detail/jRCT2053190081)
Endocrinology	CiPSC-derived Islets - Tianjin First Center Hospital (ChiCTR2300072200)	Autologous CiPSCs (from adipose)	Type 1 Diabetes Mellitus	Phase I	One-year data: insulin independence, HbA1c ∼5%, no complications	Recruiting	2021/2025 (China)	Wang et al. (2024) https://www.chictr.org.cn/showprojEN.html?proj=192835
Hematology	iPLAT1 - CiRA, Kyoto Univ. (jRCTa050190117)	Autologous iPSC-derived megakaryocytes	Thrombocytopenia	Phase I	Platelets derived from iPSCs transfused in one patient; no serious AEs	Completed (1 patient)	2019/2021 (Japan)	Sugimoto et al. (2022) https://jrct.niph.go.jp/latest-detail/jRCTa050190117
Neurology	McLean Hospital - iPSC-derived DA progenitors	Autologous iPSC-derived DA progenitors	Parkinson’s disease	Phase I	Single-patient compassionate use. No AEs reported	Completed	2017/2020 (USA)	Schweitzer et al. (2020) Song et al. (2020)
	iPSC-derived Dopaminergic Neurons - Kyoto Univ. (jRCT2090220384)	Allogeneic iPSC-derived DA neurons	Parkinson’s disease	Phase I/II	Seven patients treated; motor score improvement, no tumors or serious AEs	Completed	2018/2024 (Japan)	(Sawamoto et al., 2025) https://jrct.mhlw.go.jp/en-latest-detail/jRCT2090220384
	ASPIRO - Aspen Neuroscience (NCT06344026)	Autologous iPSC-derived DA neuron precursors	Sporadic Parkinson’s disease	Phase I/IIa	Four patients were treated; the motor score improved by up to 45% at 6 months	Ongoing	2024/2030 (USA)	https://clinicaltrials.gov/study/NCT06344026
Ophthalmology	RIKEN - Masayo Takahashi (UMIN000011929)	Autologous iPSC-derived RPE cells	Wet AMD	Phase I	One patient was treated; the second was canceled due to a somatic mutation. Program discontinued	Discontinued	2014/2015 (Japan)	Mandai et al. (2017)
	iPSC-RPE Suspension - Kobe City Eye Hospital (UMIN000026003)	Allogeneic/HLA-matched iPSC-derived RPE suspension	Wet AMD	Phase I	5 patients treated; no serious AEs	Completed	2017/2019 (Japan)	Sugita et al. (2020)
	aiPSC-RPE - VCCT Inc. (jRCTa050210178)	HLA-matched iPSC-RPE cell strip	Dry AMD/RP	Phase I/II	Subretinal delivery in 3 patients; stable vision; mild immune reaction in 1 case	Ongoing	2022/- (Japan)	Sakai et al. (2025)
	iPSC-RPE/PLGA - NIH/NEI (NCT04339764)	Autologous iPSC-RPE on PLGA scaffold	Geographic Atrophy	Phase I/IIa	20 planned; 1-year safety and 5-year efficacy endpoints	Ongoing	2020/2029 (USA)	https://clinicaltrials.gov/study/NCT04339764
	STREAM - Hospital of 15-20, Paris (NCT03963154)	hESC-derived RPE patch	Retinitis pigmentosa	Phase I/II	7 of 12 patients enrolled; long-term follow-up ongoing	Active	2019/2026 (est.) (France)	https://clinicaltrials.gov/study/NCT03963154
	Retinal Organoid Sheets - CiRA (jRCTa050200027)	Allogeneic iPSC-derived retinal organoid sheets	Retinitis pigmentosa	Phase I	Sheets (0.5 × 1 mm) implanted subretinally; initial safety evaluation completed	Follow-up ongoing	2020/- (Japan)	Hirami (2003), Watari et al. (2023)
	OpCT-001 - BlueRock (NCT06789445)	Allogeneic iPSC-derived photoreceptor precursors	Photoreceptor dystrophies	Phase I/II	54 patients planned. Evaluating safety and function of subretinal delivery	Recruiting	2025/2030 (USA)	https://trialsearch.who.int/Trial2.aspx?TrialID=NCT06789445
	CLS-001 - Keio Univ. (jRCTa031210199)	Allogeneic iPSC-derived corneal endothelial cells	Bullous keratopathy	Phase I	One patient dosed via intracameral injection	Ongoing	2022/- (Japan)	Masatoshi and Mieko (2021)
	iCEPS - Osaka Univ. (UMIN000036539)	Allogeneic iPSC-derived corneal epithelial sheets	Limbal stem cell deficiency	Phase I	4 patients treated; visual improvement, no serious AEs	Completed	2019/2021 (Japan)	Soma et al. (2024) https://center6.umin.ac.jp/cgi-open-bin/ctr_e/ctr_view.cgi?recptno=R000041628
	EyeCyte-RPE - Eyestem (NCT06394232/CTRI/2023/05/052,378)	Allogeneic iPSC-derived RPE cells	Dry AMD (GA)	Phase I/IIa	India-based study; 9 patients dosed to date; early visual gains reported	Ongoing	2024/2030 (India)	Soundararajan et al. (2025) https://clinicaltrials.gov/study/NCT06394232
Oncology	FT500 - Fate Therapeutics (NCT03841110)	Allogeneic iPSC-derived NK cells	Advanced tumors and hematologic cancers	Phase I	37 patients treated; safe with ICIs; no severe toxicities	Completed	2019/2022 (USA)	https://clinicaltrials.gov/study/NCT03841110
Orthopedics	TACK-iPS - Kyoto Univ. (jRCTa050190104)	Allogeneic iPSC-derived chondrocytes (direct)	Articular cartilage damage (knee)	Pilot	Direct iPSC-to-chondrocyte; 4 patients treated; trial terminated	Terminated	2020/2021 (Japan)	https://hpscreg.eu/browse/trial/72 (https://jrct.niph.go.jp/en-latest-detail/jRCTa050190104) (Ali et al., 2024)
	Post-TACK-iPS - Kyoto Univ. (jRCT1050220051)	Allogeneic iPSC-derived cartilage	KOA	Observational	Follow-up of TACK-iPS participants for knee function and safety	Recruiting	2022/- (Japan)	https://jrct.niph.go.jp/en-latest-detail/jRCT1050220051
	SCUlpTOR - Univ. of Sydney (ACTRN12620000870954)	Allogeneic iPSC-derived MSCs (Cymerus^®^)	KOA	Phase III	440 patients; double-blind RCT assessing symptom relief and cartilage thickness	Active, not recruiting	2021/2025 (Australia)	(Liu et al., 2021) https://anzctr.org.au/Trial/Registration/TrialReview.aspx?ACTRN=12620000870954
	NCR100 - Nuwacell Biotech (NCT06049342)	Allogeneic iPSC-derived MSCs	KOA	Phase I	China-based trial evaluating intra-articular injection safety	Not yet recruiting	2024/2025 (China)	https://clinicaltrials.gov/study/NCT06049342 (Ali et al., 2024)

Abbreviations: iPSC, induced pluripotent stem cell; MSC, mesenchymal stromal cell; DA, dopaminergic; RPE, retinal pigment epithelium; AMD, age-related macular degeneration; GA, geographic atrophy; RP, retinitis pigmentosa; KOA, knee osteoarthritis; EF, ejection fraction; RCT, randomized controlled trial; GMP, good manufacturing practice; AE, adverse event.

## 4 Regulatory considerations for the clinical translation of iPSC-based therapies

As iPSC therapies near clinical use, differences in regulatory systems across regions present key challenges. This section highlights major pathways, including IND and IMPD processes, GMP standards, approved case examples, and steps toward international alignment.

### 4.1 Regional regulatory frameworks: FDA, EMA, and PMDA

In the United States, the FDA regulates most iPSC-derived products as 351 HCT/Ps under the Public Health Service Act when they are more than minimally manipulated or used for non-homologous purposes (U.S. Food and Drug Administration, 2020). These products are classified as biologics and require an Investigational New Drug (IND) application before clinical use (U.S. Food and Drug Administration, 2007). While early-stage trials do not require a separate manufacturing license, detailed chemistry, manufacturing, and control (CMC) information is essential. The FDA supports accelerated approval through programs like The RMAT designation applies to therapies intended for serious conditions and includes requirements for post-marketing safety measures, such as Risk Evaluation and Mitigation Strategies (REMS) (Hirai et al., 2023).

In the European Union (EU), iPSC-based therapies are regulated by the EMA as Advanced Therapy Medicinal Products (ATMPs) under Regulation EC No. 1394/2007. Their approval must go through the centralized procedure, and clinical trials require authorization under the Clinical Trials Regulation (CTR 536/2014) (Madrid et al., 2024).

EMA guidelines enforce ATMP-specific Good Manufacturing Practices (GMP) and require extensive data on tumorigenicity, immunogenicity, and long-term safety. Ethical standards are strict, with bans on the use of embryonic material and mandatory informed donor consent. Post-marketing safety is tracked through Risk Management Plans (RMPs) and EudraVigilance (Martins and Ribeiro, 2025).

Japan follows a hybrid regulatory approach through the Pharmaceuticals and Medical Devices Agency (PMDA). iPSC therapies fall under the PMD Act and the Act on the Safety of Regenerative Medicine (ASRM). Japan allows clinical trials to begin through prior notification, which streamlines early-phase studies (Azuma and Yamanaka, 2016). A unique feature is the conditional and time-limited approval system, which permits product use before full efficacy data is available, with extended post-marketing monitoring for up to 7 years (Song S. J. et al., 2024). Japan also limits clinical germline editing for reproductive purposes while permitting it for research purposes (Ishii, 2015; Ishii, 2017).

While each agency has its own priorities, all three participate in ongoing Although regulatory agencies differ in focus, they are involved in joint efforts to align standards. Examples include the FDA–EMA Parallel Scientific Advice (PSA) program and collaboration through the International Council for Harmonisation (ICH) (Thor et al., 2023).

### 4.2 IND and IMPD application pathways

The IND (FDA) and IMPD (EMA) submission formats share core requirements but differ in implementation. Both require comprehensive documentation on the cell source, reprogramming and differentiation protocols, and assays for identity, purity, and potency (Hirai et al., 2023). In the EU, clinical-grade manufacturing facilities must already be GMP-certified at the trial stage, while in the U.S., quality oversight is integrated into the IND process without a separate facility license (Hirai et al., 2023). Post-trial safety planning also differs: the U.S. uses REMS, while the EU mandates an RMP (Hirai et al., 2023). Japan follows similar technical requirements but allows earlier trial entry via simplified notification-based submissions (Anklam et al., 2022; Hirai et al., 2023; Su et al., 2024).

**FIGURE 3:**
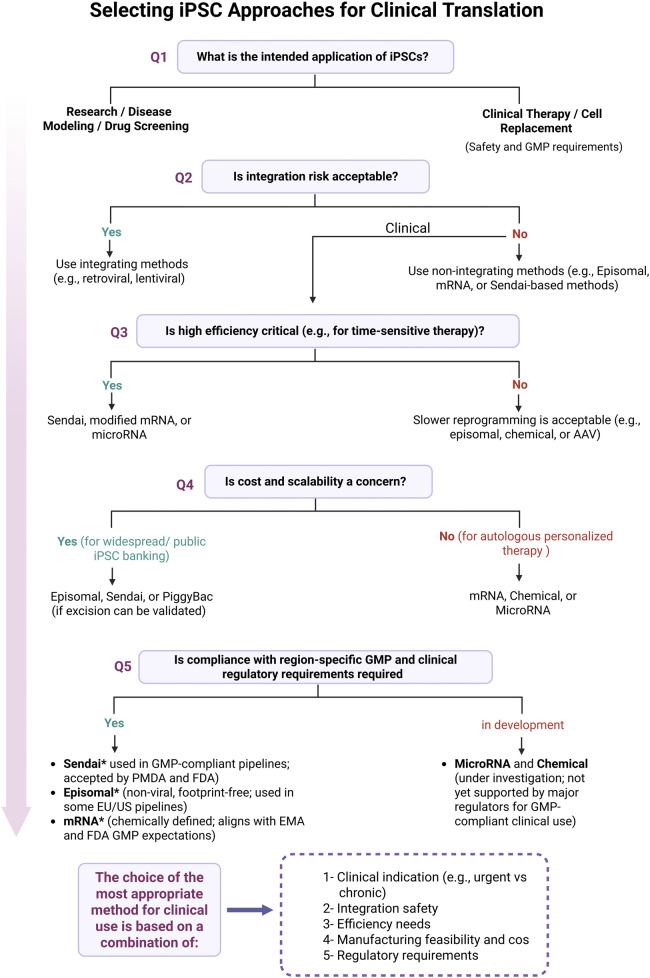
Decision-making process for selecting iPSC reprogramming strategies for clinical translation. Shown here are the key decision points involved in selecting the appropriate iPSC reprogramming method for clinical use. The flowchart starts with the intended application, research or therapy, and walks through practical considerations like integration risk, efficiency needs, scalability, and regulatory compliance. Methods such as Sendai virus, episomal vectors, and synthetic mRNA are emphasized for their current use in GMP-compliant workflows. Asterisks (*) indicate alignment with current regulatory guidance from the FDA, EMA, and PMDA, as discussed in this review. Final method selection should take into account clinical indication, integration safety, manufacturing feasibility, and region-specific regulatory requirements. Figure generated using BioRender.com.

Figure 3 illustrates how these regulatory differences guide the selection of reprogramming methods based on clinical application, safety, efficiency, and scalability.

### 4.3 Regional GMP requirements

Regulatory standards for iPSC therapy manufacturing are still developing and vary between regions. In Europe, particular emphasis is placed on aseptic processing, batch-to-batch consistency, and complete traceability, especially important due to the absence of terminal sterilization option (Martins and Ribeiro, 2025). The FDA places growing emphasis on in-process controls, raw material standards, and comparability between manufacturing runs, particularly in xeno-free, feeder-free systems (Anklam et al., 2022; Hirai et al., 2023). Japan’s regulatory framework is guided by a risk-based philosophy, placing strong importance on tracking the full history of each cell line, confirming the reliability of master cell banks, and monitoring essential quality features throughout production (Azuma and Yamanaka, 2016). Though the specifics vary by region, the overarching focus remains the same: producing safe, consistent therapies and preventing tumor-related risks.

### 4.4 Case studies of regulatory approvals

A number of early clinical trials show how iPSC-based therapies are starting to enter real-world treatment pathways. In Japan, the Kyoto Trial involved transplanting iPSC-derived dopaminergic progenitors from healthy donors into Parkinson’s patients. Approved conditionally by the PMDA, the trial showed improved motor function with no evidence of tumor formation (Sawamoto et al., 2025; Takahashi et al., 2025). In a similar effort, the RIKEN trial used autologous iPSC-derived RPE sheets to treat macular degeneration, reporting no serious side effects during long-term follow-up (Mandai et al., 2017).

In the U.S., Fate Therapeutics received IND clearance and RMAT designation for iPSC-derived NK (FT500) and CAR-T (FT819) therapies, supporting early entry into trials for solid tumors and autoimmune disease (Fate Therapeutics, 2019; Hong et al., 2020; Fate Therapeutics, 2025). Gameto’s Fertilo, an iPSC-derived ovarian support cell therapy, became the first iPSC product to enter Phase III trials in the U.S. (Bruna et al., 2025).

In Europe, while no iPSC-based product has received full marketing approval, several trials are progressing under EMA oversight (Song S. J. et al., 2024). EBiSC and HipSci provide GMP-grade iPSC lines under defined protocols (Kim et al., 2019; Mah et al., 2023). In 2025, OpCT-001, an iPSC-derived photoreceptor therapy for retinal disease, received Fast Track status from the FDA (HPSC, 2024). Additionally, XellSmart Biomedical launched iPSC-based neural progenitor trials for ALS and Parkinson’s disease in both the U.S. and Asia following FDA IND clearance (Svendsen and Svendsen, 2024; XellSmart Biomedical Co, 2025).

### 4.5 International harmonization and ICH guidelines

Although no ICH guideline is specific to iPSC therapies, regulators apply modified versions of existing frameworks, including ICH Q5D (cell substrates), S6 (R1) (preclinical safety), E6 (R2) (Good Clinical Practice), and Q12 (product lifecycle management) (MC, 2023; Soares and Ribeiro, 2024). Interpretation and implementation vary by region, contributing to differences in safety and quality expectations (Hirai et al., 2023; Selfa Aspiroz et al., 2025).

Several international organizations, including ISSCR, ISCT, and GAiT, are working to define standardized criteria for assessing potency, tumor risk, and genomic integrity in iPSC-based products (Sullivan et al., 2020; Turner, 2021; Song H. W. et al., 2024). Tools like gastruloids are also being evaluated for use in reproductive toxicity testing under ICH S5 (R3) (S, 2021). Japan’s alignment with ICH principles and participation in regulatory dialogues is further accelerating convergence (N., 2003; Medical, 2025). As more clinical data becomes available, dedicated ICH guidance for iPSC-based products is expected to emerge, supporting safer and more streamlined global development.

## 5 Autologous and allogeneic iPSCs therapies

iPSC-based treatments use either cells from the patient or from unrelated donor’s therapies (Abraham et al., 2018; Cerneckis et al., 2024). This choice affects both manufacturing and immune compatibility (Cerneckis et al., 2024).

### 5.1 Autologous iPSC therapies

**FIGURE 4:**
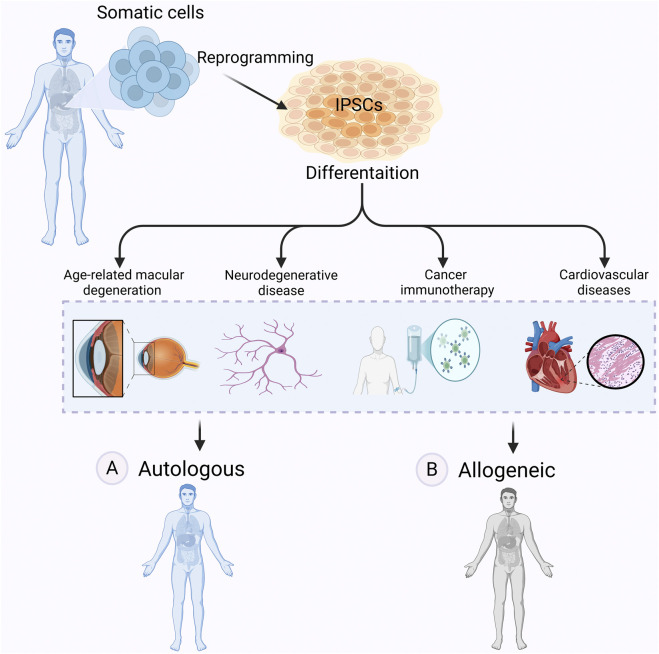
Autologous vs allogeneic iPSCs-based therapy. Autologous and allogeneic iPSC-based therapies offer distinct approaches to regenerative medicine. In autologous therapy **(A)** iPSCs are derived from a patient’s own somatic cells, differentiated into therapeutic cell types, and transplanted back into the same individual, reducing the risk of immune rejection. However, in allogeneic therapy **(B)** iPSCs are generated from a donor, differentiated, and transplanted into another patient, allowing for off-the-shelf treatments but potentially requiring immunosuppression. These therapies hold promise for treating many diseases, including age-related macular degeneration (AMD), neurodegenerative diseases, cancer, and cardiovascular diseases. Figure generated using BioRender.com.

Autologous approaches involve reprogramming a patient’s own somatic cells into iPSCs, followed by differentiation into the required cell type for transplantation (Scheiner et al., 2014; Mandai et al., 2017; Sugimoto et al., 2022) (Figure 4A). A key advantage is immune compatibility: since the cells originate from the patient, they are unlikely to be rejected and do not cause graft-versus-host disease (GvHD) (Morizane et al., 2013). Long-term immunosuppression is generally not needed (Cerneckis et al., 2024).

Parkinson’s disease is among the most studied targets for autologous iPSC-based therapy. Patient-derived dopaminergic neurons are being developed to replace lost cells and restore motor function (Hallett et al., 2015). While this approach reduces immune risk, it is time-consuming and technically demanding (Morizane et al., 2013; Madrid et al., 2021). The time required for reprogramming and differentiation, often several months, makes autologous iPSC therapies unsuitable for acute conditions like stroke or myocardial infarction (Fujimori et al., 2017; Madrid et al., 2021; Yan et al., 2024). Because each product is patient-specific, the process is labour-intensive and expensive, and results can vary from one batch to another (Cha et al., 2023).

Genetic defects present in the patient’s cells may also carry over into the iPSC-derived cells, which could compromise the intended therapeutic effect (Liang et al., 2020; Wang et al., 2020; Cerneckis et al., 2024). In such cases, genetic screening and correction, when feasible, may be needed prior to transplantation (Madrid et al., 2021).

### 5.2 Allogeneic iPSC therapies

Allogeneic approaches rely on iPSC lines derived from healthy donors (McKenna and Perlingeiro, 2023) (Figure 4B). These cell lines can be expanded and banked in advance, allowing off-the-shelf use. A shared source also simplifies manufacturing and reduces production costs by eliminating the need to generate patient-specific lines.

A major limitation is immune compatibility. Since donor cells are not matched to the recipient, they may be recognized as foreign and rejected (Sasaki et al., 2015). To prevent this, patients typically require immunosuppressive therapy required (McKenna and Perlingeiro, 2023). Extended use increases the risk of infection and may lead to metabolic or cardiovascular side effects (McKenna and Perlingeiro, 2023).

One strategy to reduce rejection involves using iPSC lines from donors with common HLA haplotypes, allowing partial matching across broader patient groups. For example, a 100-line HLA-matched iPSC bank could cover an estimated 78% of European Americans, 52% of Hispanics, and 45% of African Americans (Garreta et al., 2018). Although this approach improves compatibility, short-term immunosuppression may still be required, particularly for transplants in immune-privileged sites such as the brain or eye (Taylor et al., 2012).

**FIGURE 5:**
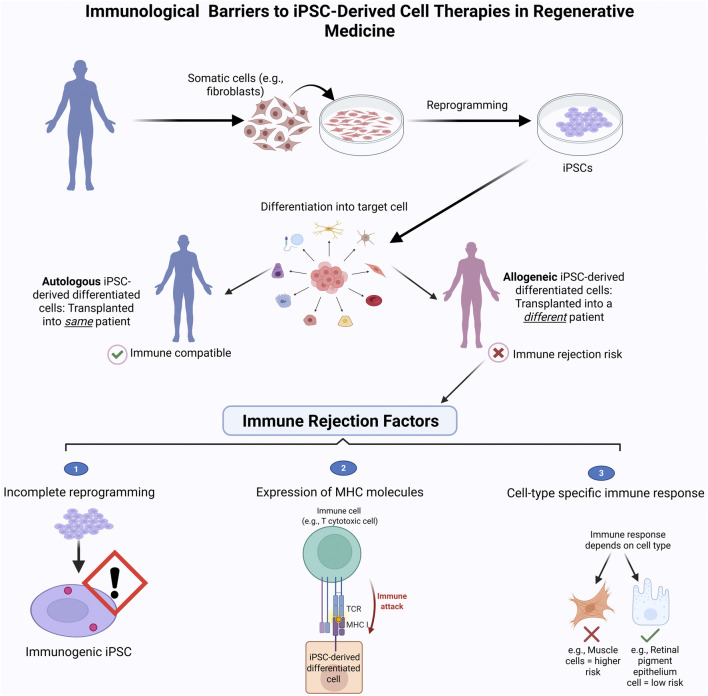
Immune Rejection challenges in iPSC-based therapies. This schematic compares the clinical workflow and immune compatibility of autologous (patient-derived) and allogeneic (donor-derived) induced pluripotent stem cell (iPSC) therapies. Autologous iPSCs are generated from the patient’s own somatic cells, minimizing the risk of immune rejection. In contrast, allogeneic iPSCs are derived from unrelated donors and may trigger immune responses, requiring immunosuppression. The bottom panel highlights key immune rejection factors: (1) expression of immunogenic markers on undifferentiated cells, (2) activation of T-cell-mediated immune attack due to mismatched MHC molecules, and (3) variable immunogenicity across different iPSC-derived cell types. Strategies such as immune-evasive engineering or use of HLA-matched iPSC banks aim to reduce these barriers and enhance the safety of allogeneic therapies. Figure generated using BioRender.com.

Figure 5 highlights key differences between autologous and allogeneic iPSC-based therapies and summarizes factors contributing to immune rejection in clinical settings.

## 6 HLA matched iPSCs banks

To further address the issue of immune rejection, researchers have established HLA-matched iPSC banks to provide readily available iPSC-derived cells that are genetically compatible with a wide portion of the population (Bradley et al., 2002; Taylor et al., 2011). These banks consist of iPSC lines derived from carefully selected donors who are homozygous for common HLA haplotypes, which increases the likelihood of finding suitable matches for recipients and reduces the need for immunosuppressive therapy One study showed that a haplobank containing 150 iPSC lines from HLA-homozygous donors could provide a suitable match for up to 93% of the UK population, highlighting the potential of this approach to improve accessibility and reduce immunogenicity in allogeneic iPSC therapies (Taylor et al., 2012).

Growing HLA-matched iPSC banks around the world could make iPSC therapies more useful in the clinic by improving immune compatibility for a wider range of patients. Over time, these haplobanks may form the backbone of future regenerative treatments (Taylor et al., 2012).

## 7 Challenges for the clinical application of iPSC-based therapies

Although iPSC-based therapies are advancing, several key barriers still limit their clinical use. Key among these are concerns about genomic stability, tumor risk, residual epigenetic memory, manufacturing scalability, quality assurance, cost, and production timelines; factors that must be addressed before these therapies can be widely implemented (Wei et al., 2024).

### 7.1 Safety concerns

#### 7.1.1 Genetic instability and tumorigenicity

Reprogramming somatic cells into iPSCs and expanding them in culture can lead to the emergence of genetic and chromosomal abnormalities. These changes are frequently linked to incomplete epigenetic reconfiguration during the acquisition of pluripotency and continue to remain a significant obstacle to moving iPSCs into routine clinical use (Rowe and Daley, 2019). In a clinical trial for age-related macular degeneration, genetic abnormalities were found in both the reprogrammed iPSCs and the derived retinal pigment epithelial cells, leading investigators to withdraw a second patient from the study (Mandai et al., 2017).

Repeated passaging of iPSCs can increase the accumulation of mutations, some of which may persist in the differentiated progeny. In recent years, researchers have uncovered a number of genetic red flags in iPSCs that raise important safety concerns (Kim et al., 2017; Merkle et al., 2017). For example, mutations in well-known cancer-associated genes like *TP53* have been detected through exome sequencing in some iPSC lines, echoing similar findings reported in mesenchymal stem cells (Kim et al., 2017; Merkle et al., 2017). To reduce the risk of tumor formation, it is critical that iPSCs are fully differentiated before transplantation (Liu et al., 2013). Even low numbers of undifferentiated iPSCs have been shown to form teratomas *in vivo*, highlighting the importance of complete differentiation and sensitive detection prior to clinical use (Gutierrez-Aranda et al., 2010; Gropp et al., 2012).

Various methods have been tested to eliminate residual undifferentiated iPSCs, including magnetic bead sorting, flow cytometry, and the use of small molecules that selectively target pluripotent cells (Doss and Sachinidis, 2019). More recently, label-free microfluidic approaches based on differences in cell size and mechanical properties have been used to deplete *OCT4*-positive cells while preserving viability, offering a scalable alternative for clinical workflows (Nguyen et al., 2024). While these strategies have shown potential, results have been inconsistent across settings, and reproducibility remains a challenge. There is still a need for more sensitive and reliable assays to detect rare undifferentiated cells and evaluate tumorigenic risk with sufficient precision.

**FIGURE 6:**
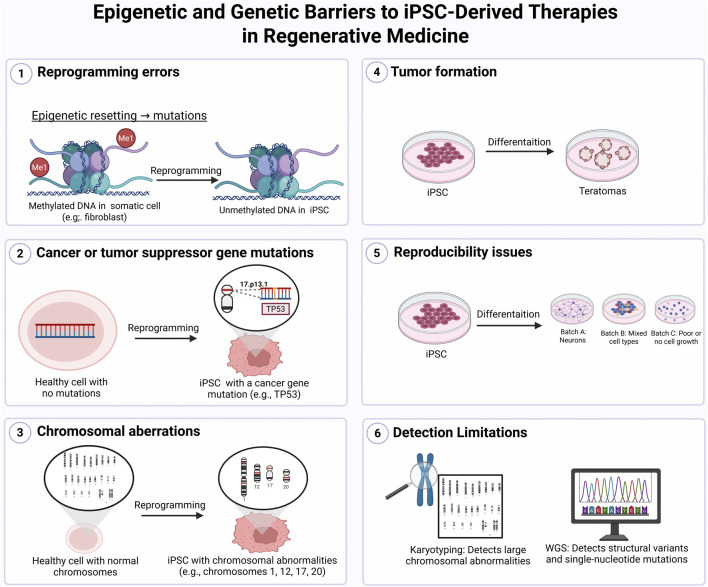
Epigenetic and Genetic Barriers to iPSC-Derived Therapies in Regenerative Medicine. Six major limitations affect the clinical translation of induced pluripotent stem cells (iPSCs): (1) epigenetic reprogramming errors that can introduce mutations, (2) cancer-related gene mutations such as TP53 arising during or after reprogramming, (3) chromosomal abnormalities including recurrent alterations in chromosomes 1, 12, 17, and 20, (4) risk of tumor formation from undifferentiated or partially differentiated iPSCs, (5) reproducibility issues across different batches, and (6) limitations in detection methods, where karyotyping may miss small changes detectable by whole-genome sequencing (WGS). These challenges emphasize the importance of rigorous genetic and epigenetic screening in clinical-grade iPSC production. Figure generated using BioRender.com.

In addition to risks posed by residual undifferentiated cells, chromosomal instability remains one of the key obstacles in advancing iPSC-based therapies toward clinical use. Certain chromosomes, notably 1, 12, 17, and 20, are especially prone to acquiring recurrent mutations over time in culture (Laurent et al., 2011). In culture, certain mutations may offer a growth advantage, allowing affected clones to gradually dominate the population. Such clonal drift contributes to variable differentiation outcomes and inter-line inconsistencies (Moy et al., 2023). These disruptions are further amplified by the cumulative stress imposed by reprogramming and extended passaging. Genomic integrity may be further compromised by oxidative stress encountered during early reprogramming or expansion phases, and the inclusion of oncogenes like *c-MYC* in certain protocols has been shown to elevate this risk (Turinetto et al., 2017). Such alterations reduce consistency and raise important safety concerns for sustained therapeutic use. These issues are summarized in Figure 6, which illustrates the major genetic and epigenetic barriers currently limiting iPSC-based therapies.

#### 7.1.2 Epigenetic memory and immune rejection

Although iPSCs derived from a patient’s own cells are often expected to avoid immune rejection, this is not always the case. In some instances, incomplete reprogramming or the abnormal expression of immunogenic proteins can still trigger immune responses after transplantation (Moquin-Beaudry et al., 2022; Chehelgerdi et al., 2023). These findings highlight the need to carefully evaluate the genetic and immunological properties of iPSC lines before they are used clinically.

Even with immune compatibility in place, additional biological hurdles remain. Transplanted cells must not only survive but also establish stable, functional connections with host tissues and complete their maturation into the appropriate cell type. Ensuring this happens reliably depends on the quality of differentiation protocols and the effectiveness of engraftment techniques (Fang et al., 2020). At the same time, broader ethical considerations, including informed consent, data privacy, and equitable access, remain central to the responsible advancement of iPSC-based therapies (Orzechowski et al., 2021; Chehelgerdi et al., 2023).

Preclinical studies have also raised important questions about the immunogenicity of autologous iPSCs. For example, Zhao et al. (2015) found that undifferentiated iPSCs elicited an immune response in humanized mice, likely due to atypical expression of embryonic or stress-related antigens (Zhao et al., 2015). Even in allogeneic settings, matching donor and recipient HLA profiles reduces, but does not eliminate, the risk of immune rejection. One explanation lies in minor histocompatibility antigens (miHAs): peptide fragments derived from intracellular proteins that vary among individuals and can provoke a T cell response even in HLA-matched transplants (Taylor et al., 2012). These peptides may become more prominently expressed during differentiation, further increasing the risk.

To address these challenges, several groups have proposed creating iPSC banks from HLA-homozygous donors. Based on population modeling, a collection of around 150 lines could match over 90% of individuals in the UK (Taylor et al., 2012) Still, even with optimal HLA matching, miHA mismatches can remain a problem. In such cases, short-term immunosuppression may still be necessary, highlighting the limitations of HLA matching as a stand-alone strategy.

Building on this, researchers are turning to immune engineering. Deuse et al. (2019) developed iPSCs with deleted MHC class I and II genes and overexpression of *CD47*, which allowed them to evade immune detection in fully immunocompetent mice (Deuse et al., 2019). In a complementary strategy, Tsuneyoshi et al. (2024) engineered human iPSCs to express key immune-modulatory proteins, *HLA-G*, *PD-L1*, and *PD-L2*, resulting in effective suppression of both T cell and NK cell responses. Taken together, such immune (Tsuneyoshi et al., 2024). Taken together, such immune engineering approaches, when combined with HLA-matching strategies, could help pave the way toward more broadly compatible and clinically viable iPSC-based therapies.

In addition to genetic engineering of classical immune markers, other approaches are being investigated to promote tolerance. Molecules such as PD-L1, indoleamine 2,3-dioxygenase (IDO), and *galectin-1* have been shown to suppress T cell activation and shift immune responses toward tolerance (Perillo et al., 1995; Riella et al., 2011; Cedeno-Laurent et al., 2012; Murata et al., 2020; Tsuneyoshi et al., 2024). When used in combination with temporary immunosuppressive regimens or tolerance-induction protocols, these strategies may help further minimiz e rejection risk (Murata et al., 2020).

Another factor that may complicate iPSC behavior is epigenetic memory. Ideally, the reprogramming process should erase the donor cell’s original epigenetic landscape, including DNA methylation, histone modifications, and regulatory RNAs, and replace it with a pluripotent identity. In reality, this reset is often incomplete (Pellegrini et al., 2022). Residual epigenetic features from the donor cell type can bias iPSCs toward their original lineage (Pellegrini et al., 2022). For instance, iPSCs generated from pancreatic β-cells, often retain a tendency to differentiate back into insulin-producing cells (Pellegrini et al., 2022). While this can be useful in certain therapeutic settings, such lineage bias may also introduce variability that complicates standardization and raises concerns about safety. To improve the consistency and clinical reliability of iPSC-based therapies, it’s important to better understand how residual epigenetic memory influences differentiation behavior (Lister et al., 2011; Pellegrini et al., 2022).

### 7.2 Scalability and quality control

Scaling iPSC production for clinical use remains difficult. Standard 2D cultures, though useful for research, are labor-intensive and poorly suited for consistent, large-scale manufacturing. As a result, there is growing interest in suspension-based cultures and bioreactors, which enable higher cell yields and offer greater control over growth conditions (Cuesta-Gomez et al., 2023; Yehya et al., 2024).

What makes this more challenging is the sensitivity of iPSCs to even small changes in culture conditions. Maintaining uniform pluripotency and genetic stability across large batches is difficult. Variants can emerge during expansion, some minor, others more significant, compromising both safety and function (Liang and Zhang, 2013; Andrews et al., 2017; Yang et al., 2024). The persistence of undifferentiated cells, unintended lineage specification, and chromosomal abnormalities can all increase the risk of tumor formation, making stringent quality control essential (Liang et al., 2013; Takei et al., 2020; Zhong et al., 2022).

Bioreactor and 3D suspension platforms have improved scalability and reduced batch-to-batch variation (Cuesta-Gomez et al., 2023). Still achieving uniform quality across various iPSC production systems remains challenging (Mamaeva et al., 2022). However, tools such as single-cell transcriptomics, live-cell imaging, and high-throughput screening have significantly advanced real-time tracking of differentiation processes and genomic stability (Huang et al., 2017; Wu et al., 2022; Nourreddine et al., 2024). Yet, no unified global criteria exist for what qualifies as a clinically acceptable iPSC product, an ongoing challenge for both regulatory alignment and broader clinical implementation (Song S. J. et al., 2024).

Alongside scale-up challenges, iPSC manufacturing for therapeutic use must also meet the specific GMP regulations set by each region. In the United States, the Food and Drug Administration (FDA) regulates iPSC-based therapies as human cells, tissues, and cellular and tissue-based products (HCT/Ps), under 21 CFR Parts 210, 211, and 1,271. This includes donor screening, validated processes, product testing, and submission of safety data before Investigational New Drug (IND) approval is granted (Jha et al., 2021).

In Europe, the EMA designates iPSC therapies as Advanced Therapy Medicinal Products (ATMPs), subject to centralized review, detailed traceability, and compliance with EU GMP guidelines, including Annexes 2 and 13 (De Sousa et al., 2017b).

Japan has adopted a more flexible framework. In 2014, the PMDA introduced a conditional, time-limited approval system that allows regenerative therapies, including iPSC-based products, to enter clinical use based on early-phase safety and efficacy data, with continued post-market surveillance (Sipp et al., 2018).

These regulatory differences not only affect the speed and cost of clinical translation but also complicate global standardization and equitable access to iPSC-based therapies.

### 7.3 Cost and time constraints

Developing iPSC-based therapies is both time-consuming and expensive (Madrid et al., 2021; McKenna and Perlingeiro, 2023). The generation of a patient-specific iPSC line under Good Manufacturing Practice (GMP) conditions can take several months and cost more than $100,000 (Jha et al., 2021; Madrid et al., 2024). This estimated cost includes donor eligibility testing, reprogramming using non-integrating GMP-grade vectors, establishment of a master cell bank, and comprehensive release testing for sterility, identity, karyotypic stability, and pluripotency markers under current GMP standards (McKenna and Perlingeiro, 2023). It also accounts for documentation, facility overhead, and regulatory compliance.

In addition to these baseline expenses, the overall cost differs depending on the application. Autologous iPSC lines made for individual patients require custom production, which is more expensive than shared allogeneic lines. Drug screening and disease modeling are less demanding, since they do not require GMP conditions. In Japan, centralized production and access to HLA haplobanks help keep costs lower. In the United States and Europe, production is less centralized, and regulatory processes are more rigid. This, along with patient-specific workflows, makes manufacturing slower and more expensive (Jha et al., 2021; Madrid et al., 2024). This does not include the time and resources needed for differentiation and quality testing (McKenna and Perlingeiro, 2023). Autologous iPSC therapy is not suitable for acute conditions like stroke or myocardial infarction. Cost, infrastructure, and regulation remain obstacles to clinical use (Madrid et al., 2024).

### 7.4 Accessibility and equity in iPSC therapies

A key ethical concern in iPSC-based therapy is how access will be handled. Generating clinical-grade lines is costly, technically demanding, and requires trained staff and facilities that are not available in all settings. These factors make it difficult to scale the technology in a way that benefits all patient populations equally, potentially worsening existing disparities in healthcare access (Zheng, 2016; Volarevic et al., 2018; Moradi et al., 2019).

Efforts are underway to address this gap. Efforts are underway to improve how iPSC-based therapies are produced at scale and to make manufacturing more cost-effective, with support from public and non-profit sectors (Huang et al., 2019).

Recent techno-economic studies suggest that automation and scalable manufacturing platforms could reduce labor costs and improve reproducibility, helping expand access in the long term (Nießing et al., 2021; Kuebler et al., 2023). At the same time, regulatory and policy discussions have focused on how to ensure fair access and avoid restricting these therapies to only those with financial or institutional advantage (Isasi and Knoppers, 2011).

For instance, the European Bank for induced pluripotent Stem Cells (EBiSC) is a non-profit repository that provides researchers with access to a wide range of iPSC lines, promoting equitable availability of these resources (De Sousa et al., 2017a; De Sousa et al., 2017b; Huang et al., 2019; Steeg et al., 2020; Mah et al., 2023).

### 7.5 Ethical considerations of HLA banks and gene editing technologies

Using human iPSCs in the clinic brings a number of ethical concerns to light, especially when it comes to informed consent, protecting personal genetic information, and ensuring treatments are fairly accessible. Although HLA-matched iPSC banks make allogeneic therapies more practical, they also demand careful handling of donor privacy and data protection. Donors must be fully informed not only about somatic cell reprogramming but also the potential long-term use, sharing, and modification of their iPSC lines in clinical and research settings (Lowenthal et al., 2012; McCaughey et al., 2016). The risk of reidentification from genomic data further underscores the importance of compliance with international privacy regulations such as GDPR and HIPAA.

Gene-editing tools like CRISPR-Cas9, while promising for correcting mutations in iPSCs, raise concerns about unintended edits, long-term effects, and misuse. Although iPSCs are not used for germline editing, the He Jiankui case, involving the birth of gene-edited children, highlighted the need for strict ethical oversight in clinical gene editing (Greely, 2019; Guo et al., 2023). Although iPSC editing is confined to somatic cells and considered reversible, it still employs the same tools used in germline modification, reinforcing the need for clear ethical limits (Greely, 2019; Guo et al., 2023).

It's still difficult to ensure that iPSC therapies are available to everyone, since making clinical-grade cells requires equipment and expertise that many places simply do not have (Zheng, 2016; Moradi et al., 2019). One way to close this gap is by supporting public biobanks and non-profit groups like EBiSC, which help make these therapies more fairly and widely accessible (De Sousa et al., 2017b; Mah et al., 2023).

## 8 Future directions in iPSC technology: artificial intelligence and personalized medicine

Machine learning has begun to play a practical role in improving several steps of iPSC-based research. Dobner et al. (2024) developed hiPSCore, a scoring system that uses gene expression data to classify pluripotent versus differentiated cells and predict their performance in differentiation assays (Dobner et al., 2024). Yang et al. (2023) trained image-based models on live-cell morphology to detect early signs of abnormal differentiation in cardiomyocyte cultures (Yang et al., 2023). Earlier work by Joutsijoki et al. (2016) used colony morphology and support vector machines to automate quality assessment in iPSC cultures (Joutsijoki et al., 2016). Marzec-Schmidt et al. (2023) trained a model on imaging data from hepatocyte differentiation and used it to classify cells based on the developmental stage. The system worked without molecular markers and matched well with experimental validation (Marzec-Schmidt et al., 2023).

Patient-specific iPSCs have also provided a useful platform for studying disease mechanisms in a genetic background that reflects individual variation (Paik et al., 2020). In neurodegenerative models, including Parkinson’s and Alzheimer’s disease, iPSC-derived cells have revealed molecular changes not detectable in traditional systems (Valadez-Barba et al., 2020). Autologous iPSCs, generated from the patient’s own cells, are being investigated as a way to avoid immune rejection and reduce the need for immunosuppressive treatment (Madrid et al., 2021). However, limitations related to scalability, quality control, and regulatory compliance remain significant hurdles for broader clinical use (Neofytou et al., 2015; Jha et al., 2021).

AI tools are now being used to support iPSC workflows. For example, convolutional neural networks (CNNs) have been used to classify colony morphology, helping assess colony quality more consistently across users (Mamaeva et al., 2022). In iPSC-based drug studies, machine learning has been used to predict individual drug responses and identify phenotypic subgroups in cardiovascular models (Paik et al., 2020).

Genome editing tools now enable single-nucleotide changes in iPSCs. Base editors can convert C to T or A to G without introducing double-strand breaks. Engineered deaminases linked to inactive Cas9 have been paired with enrichment tools like BIG-TREE to increase precision and editing efficiency in hPSCs (Tekel et al., 2021).

Prime editing offers even greater flexibility. By combining a Cas9 nickase with a reverse transcriptase and a prime editing guide RNA (pegRNA), this system supports precise base substitutions, small insertions, and deletions, all without the need for donor DNA or double-strand cleavage (Anzalone et al., 2019). Initial applications in patient-derived iPSCs using mRNA delivery have shown efficient, scarless genetic corrections (Sürün et al., 2020).

More recently, Cerna-Chavez et al. (2024) developed a robust prime editing protocol in human iPSCs to generate isogenic models of Mendelian diseases, achieving editing efficiencies as high as 73% in genes such as *NMNAT1*, *PRPF3*, and *PRPF8*. In parallel, Wu et al., 2024a introduced an all-in-one inducible system, PE-Plus, which enables multiplex and temporally controlled edits in pluripotent stem cells with enhanced specificity and minimal off-target activity (Cerna-Chavez et al., 2024; Wu Y. et al., 2024).

To support cell survival and expansion after genome editing, researchers have developed post-editing support strategies. One example is the CEPT cocktail, a chemically defined formulation containing chroman 1, emricasan, polyamines, and trans-ISRIB, which promotes clonal expansion and survival of single iPSCs following stress-inducing procedures like dissociation or editing (Tristan et al., 2023).

Collectively, the advances in AI, gene editing, and cell purification are contributing to the development of more clinically viable, personalized iPSC therapies.

## 9 Concluding remarks

The use of iPSCs in regenerative medicine has brought hope for patient-specific treatments, but their clinical application still faces major challenges (Chehelgerdi et al., 2023; Cerneckis et al., 2024). Issues such as genetic instability, tumorigenic risk, immune rejection, and large-scale production obstacles must be addressed before these therapies become widely available (Yamanaka, 2020; Moy et al., 2023). Advances such as gene editing, optimization of cell differentiation protocols, and the development of HLA-matched iPSC banks have helped to overcome some of these challenges (Kitano et al., 2022; Alowaysi et al., 2023). Future research will focus on improving reprogramming and differentiation protocols, long-term safety, and integrating newer technologies to enhance efficacy and make iPSC-based treatments more practical and broadly accessible (Cerneckis et al., 2024). The coming decade will reveal whether these technologies can move from highly controlled trial settings into routine practice, a transition that will define the true clinical impact of iPSCs.
