# Supplementary material for: Clinical translation of human iPSC technologies: advances, safety concerns, and future directions
Source: Front Cell Dev Biol. 2025 Sep 22;13:1627149. doi: 10.3389/fcell.2025.1627149 (PMC12498231; doi:10.3389/fcell.2025.1627149)
Supplement: Supplementary file 1 [file Table1.docx]

**Table S1. Summary of iPSC reprogramming methods.**

This table presents a comparative overview of commonly employed techniques for generating induced pluripotent stem cells (iPSCs). It outlines each method’s integration profile, reprogramming efficiency, approximate timeframe for colony emergence, principal features, and associated limitations. Together, these comparisons provide context for selecting an appropriate reprogramming strategy based on safety, efficiency, and translational potential.

| **Method** | **Integration** | **Efficiency (%)** | **Colony Emergence (days)** | **Key Features** | **Limitations** | **References** |
| --- | --- | --- | --- | --- | --- | --- |
| Retroviral/Lentiviral Vectors | Yes | 0.02-0.08/ 0.02-1% | 25-30 | High efficiency; risk of insertional mutagenesis | Insertional mutagenesis; transgene reactivation | (Rao and Malik, 2012) |
| Adenovirus | No | 0.0001-0.001 | 12-16 | High transduction efficiency;high load capacity; temporary gene expression | Repeated transduction required; immunogenicity; low efficiency | (Zhou and Freed, 2009; Haridhasapavalan et al., 2019) |
| Adeno-associated virus | No | <0.01 | 14-21 | Low immunogenecity | Very low efficiency; Small load capacity | (Hirsch et al., 2016; Martino and Markusic, 2020; Scesa et al., 2021) |
| Sendai Virus (SeV) | No | 0.01- 1 | 10-25 | High efficiency; cytoplasmic; cleared by ~P10 | Requires viral handling; genome clearance verification | (Seki et al., 2010; Beers et al., 2015; Schlaeger et al., 2015; Okumura et al., 2019; Scesa et al., 2021). |
| PiggyBac Transposon | Yes (reversible) | 0.02-0.1 | 10-14 | Single transfection; precise excision; reintegration risk | Reintegration risk; requires validation of excision | (Kaji et al., 2009; Woltjen et al., 2009; Mali et al., 2010) |
| Sleeping Beauty Transposon | Yes (reversible) | ~0.1 | 6-18 | Fast colony emergence; random integration; SB100X variant | Random integration; limited human data | (Grabundzija et al., 2013). |
| Episomal Vectors | No | 0.0003-0.03 | 25-30 | Simple; low efficiency; repeated transfection needed | Low efficiency; repeated transfection needed | (Yu et al., 2007; Scesa et al., 2021). |
| Minicircle Vectors | No | ~0.005 | 14-18 | Improved vector; better stability; still low efficiency | Still low efficiency; difficult scalability | (Narsinh et al., 2011; Scesa et al., 2021). |
| Modified mRNA | No | 1.4-4.4 | 15-20 | High efficiency; non-integrating; requires daily transfection | Labor-intensive; immunogenicity risk; expensive | (Warren et al., 2010; Wang, 2021) |
| MicroRNA | No | Up to 10 | 6-7 | Footprint-free; potent; delivery and stability challenges | Delivery challenges; transient expression; off-target effects | (Anokye-Danso et al., 2011; Liao et al., 2011; Miyoshi et al., 2011; Lipchina et al., 2012) |
| Chemical Reprogramming | No | ~0.2 (up to ~1.5 in new protocols) | 16-40 | Fully non-genetic; slower; improving with optimized protocols | Slow reprogramming; variable responsiveness; low yield | (Hou et al., 2013; Qin et al., 2017; Liuyang et al., 2023; Wang et al., 2023). |
